# Effect of Pulsed Electric Field (PEF) Pretreatments on the Phytochemical Composition, Antioxidant, and Antimicrobial Properties of Sage Extracts

**DOI:** 10.1155/ijfo/3565398

**Published:** 2026-09-23

**Authors:** Stella Plazzotta, Gian Marco Artuso, Sofia Melchior, Federico Basso, Sabrina Vorderegger, Monica Anese, Anja Schuster, Maria Concetta Tenuta, Sara Bolchini, Giovanna Ferrentino, Lara Manzocco

**Affiliations:** ^1^ Department of Agricultural, Food, Environmental and Animal Sciences, University of Udine, Udine, Italy, uniud.it; ^2^ Department of Human Sciences and Promotion of the Quality of Life, San Raffaele University, Rome, Italy, unisr.it; ^3^ Department of Health Sciences, Salzburg University of Applied Sciences, Puch bei Hallein, Austria, uni-salzburg.at; ^4^ Faculty of Agricultural, Environmental and Food Sciences, Free University of Bolzano, Bolzano, Italy, unibz.it

**Keywords:** drying, electroporation, food pathogens, herbs, polyphenols

## Abstract

This study investigated the impact of pulsed electric fields (PEFs) on the phytochemical, antioxidant, and antimicrobial properties of extracts obtained from fresh and dried sage (*Salvia officinalis* L.). Extracts were characterized in terms of dry matter, phenolic content, and further profiled using HPLC‐HRMS. Fresh sage extracts were richer in chlorophylls and chlorophyllides, whereas dried sage extracts contained significantly higher levels of total phenolics and pheophytins. Principal component analysis highlighted clear compositional differences between extracts obtained from fresh and dried sage. Although PEF had a limited impact on phenolic extraction from fresh sage, a pronounced increase was observed when applied to dried sage, likely due to electroporation facilitating solvent penetration in tissues collapsed upon drying. Antioxidant efficiency, expressed as chain‐breaking phenolic ratios (DPPH, ABTS, and FRAP), increased with PEF intensity for both matrices, with extracts from fresh sage showing the most pronounced improvement. Antimicrobial testing demonstrated selective efficacy against gram‐positive bacteria (*Bacillus cereus* and *Staphylococcus aureus*), with extracts from dried sage generally exhibiting stronger inhibition due to its higher phenolic content, whereas only the fresh sage extract treated at 8 kV/cm showed partial activity against *Pseudomonas aeruginosa*. No extract inhibited *Escherichia coli* or *Salmonella enterica* growth. Overall, PEF proved to be a valuable processing tool to enhance the extraction and functional properties of sage phytochemicals.

## 1. Introduction

Sage (*Salvia officinalis* L.) is a versatile plant of the Lamiaceae family, originating from the Mediterranean region and globally valued for its distinctive flavor and numerous culinary and medicinal benefits. The latter are due to a wide array of phytochemicals, which play crucial roles in the plant defense against herbivores, fungi, bacteria, and viruses, as well as in attracting pollinators and mediating competitive interactions with other plants [1, 2]. Among sage phytochemicals, phenolic compounds and terpenes exhibit significant bioactivity, being involved in key protective mechanisms such as oxidative stress reduction, microbial growth inhibition, and modulation of inflammation [3–5]. Previous studies confirmed the presence of rosmarinic acid, carnosic acid, carnosol quercetin, and caffeic acid in sage, which contributed to its antioxidant and antimicrobial activities [6–8]. Similar bioactivities have also been reported for sage essential oil terpenes like *α*‐thujone, camphor, and limonene [7, 9]. Based on its richness in phytochemicals, sage extracts are actually recognized as valuable resources in food, pharmaceutical, and cosmetic industries. In the food sector, sage extracts are used not only to enhance flavor and nutritional value but also serve as antioxidants [10]. Cegiełka et al. [11] used a sage extract to reduce lipid oxidation and bacterial growth during refrigerated storage of vacuum‐packed chicken meatballs. Owing to their ability to inhibit the growth of bacteria such as *Escherichia coli*, *Staphylococcus aureus*, *Bacillus cereus*, and *Salmonella typhimurium* [11, 12], sage extracts have also been incorporated into edible coatings to create protective active barriers extending packaged food shelf life [7, 10, 13].

Conventional methods for preparing plant extracts typically rely on solid–liquid extraction performed by immersing the vegetal tissue into organic solvents at high temperature [14]. Although these techniques are preferred for their simplicity, they often implicate long extraction times, high solvent consumption, and the risk of degrading heat‐sensitive phytochemicals. To increase extraction efficacy, plant tissue is usually finely ground to reduce the size of solid particles and increase the surface area exposed to the solvent. However, this process is effective only when cell compartmentalization is extensively compromised, allowing phytochemicals to migrate from within the cells into the liquid extraction solvent. For this reason, emerging technologies capable of impairing cell integrity, such as those based on ultrasounds, high‐pressure homogenization, and pulsed electric fields (PEFs), have been proposed as pretreatments to assist the extraction [15, 16].

PEFs refer to a nonthermal technology that favors the extraction of phytochemicals by increasing cell membrane permeability through electroporation phenomena [17]. The fresh plant material is ground in the presence of a solvent, generally a hydroalcoholic solution, and placed between a pair of electrodes, generating high‐voltage and short‐time (*μ*s‐ms) pulses [18]. When applied at field intensities between 0.1 and 20 kV/cm, this technology is particularly recommended for extraction assistance, as it selectively electroporates cell membranes, substantially increasing the solubilization of valuable biomolecules within liquid media [19–21]. Although previous studies have demonstrated the effectiveness of PEF‐assisted extraction of phytochemicals from various plant sources [20, 22], very scarce data are available regarding the application of the technology in the case of sage. Actually, to the best of our knowledge, only the study of Athanasiadis et al. [6] reported the application of PEF to assist the extraction of fresh sage leaves in hydroalcoholic solutions (75% *v*/*v* ethanol). In particular, these authors reported that PEF significantly increased the extractability of total and individual polyphenols, such as rosmarinic acid, while also affecting the final concentration and composition of volatile compounds of the extracts.

It is important to note that, in industrial supply chain frameworks, sage and other similar plants intended for extraction are often processed into dried leaves immediately after harvest [23]. Air‐drying is the most common method, carried out for various purposes including spoilage prevention, shelf‐life extension, concentration of phytochemicals and flavor, and improved ease of transport, storage, and market distribution. However, drying is also known to introduce unpredictable modifications in the chemical profile of plants and hence of their extracts, primarily by affecting the extractability of cellular compounds, as well as their structure, bioactivity, and stability [10, 24]. Therefore, it can be inferred that the industrial applicability of PEF‐based sage extraction would be strongly bound to the influence of drying processes on the added value of the final extracts. Nevertheless, this aspect still represents an unexplored research gap.

Based on these considerations, the present work is aimed at exploring for the first time the impact of drying on the efficacy of PEF‐assisted extraction performed on sage leaves. To this aim, hydroalcoholic homogenates of fresh and air‐dried sage were subjected to PEF‐assisted extraction at increasing intensity. The obtained extracts were analyzed for extraction yield, phytochemical composition, and antioxidant and antimicrobial activity.

## 2. Materials and Methods

### 2.1. Materials

Leaves of *S*. *officinalis* L. were provided by a local supplier. Other materials used were as follows: absolute ethanol, Folin reagent, 2,2‐diphenyl‐2‐picrylhydrazyl (DPPH), 2,2 ^′^‐azino‐bis(3‐ethylbenzothiazoline‐6‐sulfonic acid) diammonium salt, (ABTS) solution, 2,4,6‐tripyridyl‐s‐triazine (TPTZ), methanol of analytical grade, methanol and formic acid of LC‐MS grade, potassium persulfate, sodium carbonate, ferric chloride, nutrient agar, Trolox, and gallic acid (Sigma‐Aldrich, Milan, Italy); *S. aureus* (ATCC 25923), *B. cereus* (ATCC 11778), *E. coli* (ATCC 25922), *Pseudomonas aeruginosa* (*P. aeruginosa*) (ATCC 27853), *Salmonella enterica* (*S. enterica*) (ATCC 14028), Columbia agar supplemented with 5% sheep blood, Tryptic soy agar, and saline solution 0.45% (bioMérieux, Marcy‐l′Étoile, France); and UMIC Müller–Hinton II Broth (Bruker Corporation, Billerica, Massachusetts, United States), EtOH (Bruker Corporation, Billerica, Massachusetts, United States), and NaCl (bioMérieux, Marcy‐l′Étoile, France).

### 2.2. Sage Preparation

Sage leaves were processed within 2 h from harvest and divided into two aliquots. One aliquot (fresh leaves) was submitted to homogenization using a high‐speed powder grinder (HB01, Mulinex, China) set at half power for 3 min (≈0°C, ice bath), in the presence of absolute ethanol. The ratio between leaves and ethanol was equal to 0.43:1 (*w*/*w*), allowing to obtain homogenates of fresh leaves containing 10% (*w*/*w*) dry matter, 20% (*w*/*w*) water, and 70% (*w*/*w*) ethanol. The second aliquot of leaves was placed in a single layer on a stainless‐steel tray and air‐dried in a ventilated oven at 45°C for 4 days (UF55 plus, Memmert, Schwabach, Germany). At increasing times during drying, the weight of leaves was monitored to establish drying kinetics. Subsequently, the dried sage leaves were submitted to homogenization in a 10:90 (*w*/*w*) ratio with a hydroalcoholic solution using the same procedure described above for fresh leaves. The composition of the hydroalcoholic solution (ethanol:water 77.6:22.4) was established to obtain the same dry matter/water/ethanol ratio of the fresh sage homogenates.

### 2.3. Optical and Polarized‐Light Microscopy

Two drops of fresh or dried sage homogenates were poured onto a glass microscope slide and covered with a glass coverslip. Images were acquired at 20°C and 20x magnification, in optical and polarized mode, by using a Visoria B microscope (Leica Microsystems, Heerbrugg, Switzerland) equipped with a Flexacam i5 camera (Leica Microsystems). Images were saved in .jpeg format at a resolution of 3840 × 2160 pixels (4K UHD).

### 2.4. Particle Size Distribution

The particle size of the fresh and dried homogenates not subjected to PEF was measured by using a Malvern Mastersizer 3000 (Malvern Instruments Ltd., Worcestershire, United Kingdom) equipped with an Aero S dispersion system, a standard Venturi feeder, and a 2.5‐mm hopper gap. After background subtraction (measurement time: 10 s), approximately 1 mL of homogenate was collected under stirring and added dropwise to the dispersant (i.e., degassed MilliQ water) until laser obscuration reached 4.0*%* ± 0.5*%*. Size measurement was conducted in quintuple (*n* = 5) assuming nonspherical particles and with a measurement time of 10 s. Particle size distributions were expressed as volume‐based frequency distributions, reporting volume percentage (%) as a function of particle size (*μ*m).

### 2.5. PEF‐Assisted Extraction

Aliquots (13.00 ± 0.01 g) of homogenates were collected under vigorous stirring and treated in the PEF plant described by Plazzotta et al. [25], using a parallelepiped chamber (16.8 mL capacity, 1.5 cm gap). The variability attributed to ground sage sedimentation in the PEF chamber was controlled by initiating treatment within 10 s after transfer for all samples.

The input parameters (LabVIEW4PEF_B‐618‐01 9.0, ProdAl, Fisciano, Italy) included number of pulses (*n* = 2000), pulse frequency (*f* = 900 *H* *z*), and pulse width (*τ* = 5 *μ* *s*), which were kept constant for all samples. Pulse voltage (*U*_0_) was varied (0, 30, and 400 V) to obtain increasing electric field values (*E* = 0, 0.5, and 8 kV/cm, respectively) measured across the PEF cell during treatment. The intensity level corresponding to 0.5 kV/cm was specifically selected based on its frequent application to assist extraction from plant tissues in the literature [18, 26–28]. Differently, 8 kV/cm corresponded to the highest intensity treatment applicable to the homogenates without incurring arcing phenomena.

PEF‐treated samples were stirred in a beaker for 10 min at room temperature and centrifuged at 10,000 × g for 30 min at 4°C (Avanti Centrifuge J‐25, Beckman Coulter, Indianapolis, Indiana, United States). The resulting supernatant (i.e., the extract) was collected and stored at −20°C until analysis.

### 2.6. Dry Matter and Extraction Yield

A sample aliquot (0.40 ± 0.02 g) was inserted in a crucible and vacuum dried (72°C, 0.01 MPa, overnight, Vuotomatic 50, Bicasa, Milan, Italy). Dry matter (%, *w*/*w*) was calculated as the percentage ratio between the weight of the vacuum‐dried sample and the weight of the sample before drying. The extraction yield was assessed by determining the dry matter of the supernatants obtained from each PEF‐treated sample. To this aim, an aliquot of 0.40 ± 0.02 g of supernatant was weighed in a crucible, predried on a hot plate (70°C, 30 min) and analyzed for dry matter.

### 2.7. Total Phenolic Content

The method of Singleton and Rossi [29] with slight modifications was used. Amounts of 130 *μ*L of extract (0.5 mg/mL), 130 *μ*L of Folin reagent, and 130 *μ*L of saturated Na_2_CO_3_ solution were mixed with 1 mL of distilled water and the mixture was incubated in the dark for 2 h. Absorbance at 765 nm was measured (Infinite M Nano+ spectrophotometer, Tecan Italia, Milan, Italy). Analyses were performed in duplicate on two independent samples (*n* = 4), and results were expressed as milligrams of gallic acid equivalents (GAE) per 100 milligrams of dry matter using a gallic acid calibration curve (*R* ^2^ = 0.995).

### 2.8. Ultraviolet‐Visible Light Absorption Spectrum

Extracts′ UV–Vis absorbance spectra were recorded at 25°C using a UV‐2501 PC spectrophotometer (Shimadzu, Kyoto, Japan) in quartz cuvettes (1‐cm path length) with 1‐nm resolution. Samples were diluted with a hydroalcoholic solution (ethanol 80%, water 20%, *v*/*v*) at 1:500 for the UV range (200–400 nm) and 1:100 for the visible range (400–700 nm).

Spectra were normalized based on sample dilution and dry matter. Chlorophyll detection was performed by comparing the optical density normalized to extract dry matter (O.D. g_dm_^−1^) at 660 nm, as obtained from the acquired UV–Vis spectra [30].

### 2.9. Detection of Antioxidant Compounds, Chlorophylls, and Derivatives by HPLC‐HRMS

Sage extracts were characterized by high‐performance liquid chromatography coupled with high‐resolution mass spectrometry (HPLC‐HRMS). Extracts (3% *w*/*w*) were diluted 1:6 in Milli‐Q water/methanol (1:1, *v*/*v*) and filtered through 0.22‐*μ*m PTFE filters prior to analysis. Measurements were performed using an UltiMate 3000 UHPLC system coupled to a Q Exactive Orbitrap mass spectrometer (Thermo Fisher Scientific, Waltham, Massachusetts, United States). Two analytical runs were conducted for each sample to monitor antioxidant compounds and chlorophylls and their derivatives, using Trolox as internal standard. Antioxidant compounds were separated on a Kinetex Biphenyl column (Phenomenex, Torrance, California, United States) using water and methanol (both with 0.1% formic acid) as mobile phases at 0.3 mL min^−1^ and 30°C. Chlorophylls and derivatives were analyzed on an ODS‐Hypersil column (Thermo Fisher Scientific) with methanol‐based mobile phases at 1 mL min^−1^. Mass spectrometric detection was performed by electrospray ionization (ESI) with full‐scan acquisition and data‐dependent MS/MS. HPLC‐HRMS analyses were conducted on two independently prepared samples (biological replicates). Each sample was analyzed in duplicate (technical replicates), resulting in four measurements (*n* = 4). Data were processed using Chromeleon, Xcalibur, and Compound Discoverer software. Compounds were tentatively identified based on comparison of retention time (RT), HRMS/MS data, and mass fragment characteristics with compounds reported in the literature and/or available databases (PubChem, FooDB, and mzCloud). The results were expressed as area percentage of each compound compared with the total area of the compounds present in the extract. Thus, they indicated the relative abundances of the detected compounds aimed at providing a comparative characterization of the phytochemical profile of the extracts.

### 2.10. Antioxidant Activity by DPPH Assay

The antioxidant activity of sage extracts was evaluated using the DPPH radical scavenging assay following Ding et al. [31] with minor modifications. A methanolic DPPH• stock solution (2.5 mM) was diluted to 200 *μ*M to obtain the working solution. Briefly, 100 *μ*L of extract (0.5 mg mL^−1^) was mixed with 100 *μ*L of DPPH solution in a 96‐well microplate and incubated in the dark for 30 min. Absorbance was measured at 515 nm using a microplate reader (Tecan Italia, Milan, Italy). Analyses were performed in duplicate on two independent samples (*n* = 4). Radical scavenging activity was calculated as percentage inhibition according to Equation (1):

(1) %Inhibition=A0−A30A0×100,

where A_0_ is the absorbance of the DPPH control, and A_30_ is the absorbance after 30 min. Results were expressed as milligrams of Trolox equivalents (TE) per 100 mg of dry extract based on a Trolox calibration curve (*R* ^2^ = 0.998).

### 2.11. Antioxidant Activity by FRAP Assay

The reducing capacity of the extracts was determined using the FRAP assay according to Benzie and Strain [32]. The FRAP reagent was freshly prepared by mixing acetate buffer (pH 3.6), ferric chloride (20 mM), and TPTZ solution (40 mM in 40 mM HCl) in a 10:1:1 (*v*/*v*/*v*) ratio. Briefly, 10 *μ*L of extract (0.5 mg mL^−1^) was mixed with 190 *μ*L of FRAP reagent and incubated at room temperature for 30 min. Absorbance was measured at 593 nm using a microplate reader (Tecan Italia, Milan, Italy). Analyses were performed in duplicate on two independent samples (*n* = 4). Results were expressed as milligrams of TE per 100 mg of dry extract based on a Trolox calibration curve (*R* ^2^ = 0.994).

### 2.12. Antioxidant Activity by ABTS Assay

Antioxidant activity was also evaluated using the ABTS radical scavenging assay following Borlinghaus et al. [33]. The ABTS^+^ radical was generated by reacting ABTS solution (7 mM) with potassium persulfate (2.45 mM) and incubating the mixture in the dark at room temperature for 24 h. For the assay, 50 *μ*L of extract solution (0.5 mg mL^−1^) was mixed with 1 mL of ABTS^+^ solution and incubated in the dark for 10 min. Absorbance was measured at 734 nm (Tecan Italia, Milan, Italy). Analyses were performed in duplicate on two independent samples (*n* = 4). Results were expressed as milligrams of TE per 100 mg of dry extract based on a Trolox calibration curve (*R* ^2^ = 0.991).

### 2.13. Chain‐Breaking Phenolic Ratio

To compare the chain‐breaking activity of samples with different phenolic contents, the chain‐breaking phenolic ratio was calculated [34]. This ratio was obtained by dividing the antioxidant activity measured by DPPH, FRAP, and ABTS assays by the total phenolic content of the extracts.

### 2.14. Antimicrobial Activity

The bacterial panel examined in this study comprised of two gram‐positive strains, namely *B*. *cereus*, *S*. *aureus* and three gram‐negative strains, namely *E. coli*, *P. aeruginosa*, and *S. enterica*. *S. aureus* was cultured on Columbia agar supplemented with 5% sheep blood and incubated at 35°C in a CO_2_‐enriched atmosphere generated using GENbox CO_2_ systems (bioMérieux, Marcy‐l′Étoile, France). *B*. *cereus* was maintained on nutrient agar under aerobic conditions at 30°C. *P. aeruginosa*, *E. coli*, and *S. enterica* were cultivated aerobically on Tryptic soy agar at 35°C. Bacteria were revived from cryogenic stocks and inoculated by streaking onto their respective media and incubating for 24–48 h. Vitek 2 automated microbial identification system was used for identification for species identification (bioMérieux, Marcy‐l′Étoile, France). Minimum inhibitory concentrations (MICs) were determined against the bacterial strains listed above to assess the antimicrobial activity of the extracts. MIC values correspond to the lowest concentrations required to inhibit 50% (MIC_50_) or 90% (MIC_90_) of bacterial growth. The EUCAST broth microdilution protocol (Version 5.0) and the EUCAST Breakpoint Tables (Version 13.1) were followed and the protocol adapted from Gunnarsdottir et al. [35] was used. Serial dilutions of the extracts were prepared in UMIC Müller–Hinton II broth substituted with EtOH resulting in final concentrations ranging from 1600 *μ*g/mL to 6.25 *μ*g/mL (100 *μ*L per well) with an end concentration of 1% EtOH. CytoOne 96‐well microplates (Starlab International, Hamburg, Germany) were used. Bacterial suspensions were adjusted to a McFarland of 0.5 standard in 0.45% NaCl and further diluted 1:100 to yield a final concentration of 5 × 10^5^ CFU/mL. Subsequently, 50 *μ*L of the inoculum was added to each well, excluding negative control wells. In each assay, appropriate control conditions were included. Negative control wells were included containing sterile saline without bacterial inoculum to confirm sterility. Growth controls consisted of bacterial suspensions added to wells containing only culture medium with an end concentration of 1% EtOH and saline solution. Positive controls included media with an end concentration of 1% EtOH supplemented with linezolid (27 *μ*g/mL) for gram‐positive strains and ciprofloxacin (27 *μ*g/mL) for gram‐negative strains. Tecan Infinite 200 PRO plate reader (Tecan, Grödig, Austria) was used the measure the absorbance at 610 nm at 0 h and 20 h postinoculation. Growth values were normalized to the growth control. Three independent experiments were performed in triplicate on two independent samples (*n* = 6).

### 2.15. Data Analysis

Data are expressed as mean ± standard deviation. One‐way ANOVA was performed using R software (v. 3.1.1, R Foundation for Statistical Computing, Windows), followed by Tukey′s post hoc test to assess differences between means (*p* < 0.05). For analysis of MIC experiments GraphPad Prism 10.4.0 (Graphpad Software, Inc.) was used. Ordinary one‐way ANOVA followed by Dunnett′s multiple comparisons test was performed.

## 3. Results and Discussion

### 3.1. Effect of PEF on the Phytochemical Properties of Sage Extracts

**Figure 1:**
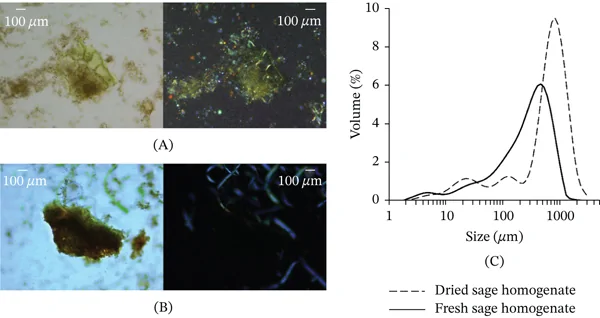
Optical and polarized‐light micrographs (20x magnification) of the homogenates obtained from (A) fresh and (B) dried sage before the application of PEF treatments. Particle size distribution (C) the samples is also shown.

In the first part of the work, the attention was focused on the characterization of the extracts obtained from fresh or dried sage without the application of PEF treatments (Controls), with particular reference to the microstructure and to the particle size distributions of the homogenates. As evidenced in Figure 1A, fresh homogenized sage presented the typical microstructure of unprocessed plant tissues, showing defined particle edges and intact cell walls. The latter were also clearly birefringent under polarized light, indicating that homogenization in the presence of ethanol did not cause substantial disruption of their cellular structure (Figure 1A). Conversely, the particles present in the homogenates obtained from dried sage were evidently collapsed, showing rough edges and highly damaged cells (i.e., indistinguishable cell walls with loss of birefringence) (Figure 1B). This was likely caused by the establishment of capillary forces at the water vapor‐liquid water interface upon air‐drying, which resulted in the disruption of the ordered three‐dimensional network of the tissue [36].

The effect of drying on sage leaves microstructure was probably the cause of the differences observed in terms of particle size. In particular, drying caused a clear shift of the size distribution towards larger diameter values (Figure 1C). This was likely due to the collapsed tissue presenting a higher mechanical strength and lower brittleness than the fresh one, being thus more difficult to disrupt by means of mechanical shearing [37].

Regardless of whether they were obtained from fresh or dried leaves and despite the differences in particle size between the two homogenates (Figure 1C), sage extracts exhibited a comparable dry matter content (~3%, *w*/*w*) (Table 1). Nevertheless, the extract from fresh sage exhibited an absorbance at 660 nm nearly three times higher than the dry sage one (Table 1), indicating a higher content of green pigments such as chlorophylls. This was reasonably due to the fact that chlorophyll would be already degraded to uncolored compounds during sage drying [38]. In contrast, the dry sage extract showed a total phenolic content substantially higher than that of the fresh extract (Table 1). Such difference was likely due to the extensive structural damage induced by drying to the sage cells (Figure 1B), which allowed for a more efficient decompartmentalization of these molecules upon extraction with the hydroalcoholic solution [39]. Based on these results, it can be hypothesized that sage extraction was influenced by the drying step primarily in terms of targeted compounds selectivity rather than quantitative yield, with the differences in particle size playing a secondary role in steering the efficaciousness of the process.

**Table 1:** Dry matter, total phenolic content, and absorbance at 660 nm of extracts obtained from fresh and dried sage by applying PEF treatments at increasing electrical field strengths (E). Controls indicate extracts not subjected to PEF.

Sample	E (kV/cm)	Dry matter (%)	Absorbance at 660 nm (O.D. g_dm_^−1^)	Total phenolic content (mg_GAE_/100 mg_d.m._)
Fresh	0 (Control)	3.16 ± 0.02^a^	4.35 ± 0.36^b^	6.6 ± 0.2^d^
	0.5	3.12 ± 0.01^a^	4.21 ± 0.17^b^	6.9 ± 0.6^d^
	8	2.49 ± 0.01^b^	6.45 ± 0.16^a^	8.6 ± 0.5^c^

Dried	0 (Control)	3.11 ± 0.09^a^	1.14 ± 0.03^c^	14.3 ± 0.4^b^
	0.5	3.24 ± 0.14^a^	1.10 ± 0.28^c^	14.8 ± 0.2^b^
	8	3.13 ± 0.05^a^	1.67 ± 0.03^c^	15.5 ± 0.5^a^

*Note:* Different letters indicate statistically different means in the same column (*p* < 0.05).

To better study the chemical profile of the extracts, HPLC‐HRMS was applied. Both extracts reported a similar phytochemical profile (Table S1). A total of 38 representative compounds were tentatively identified based on comparison of RT, HRMS/MS data, and mass fragment characteristics with compounds reported in the literature and/or available databases [40]. The results indicate the presence in the extracts of several phenolic acids, including caffeic acid and its derivatives, chlorogenic acid, salvianolic acids, ferulic acid, and rosmarinic acid. In addition to anthocyanins such as peonidin and pelargonidin, several flavonoids, including luteolin 3‐O‐D‐glucuronide and quercitrin, as well as terpenoids like carnosic acid and carnosol, were identified. These compounds were previously detected in sage extracts [41]. Analyses also revealed the presence of several compounds that contribute to the color of sage, including green pigments such as chlorophyll a and b, and their grey‐brown degradation products—pheophorbides and pheophytins [42].

To further improve the extractability of target compounds, PEF‐assisted extraction was performed on fresh and dried sage leaves. As shown in Table 1, in the case of fresh sage, the dry matter of the extracts obtained at electrical fields up to 0.5 kV/cm was similar to that obtained without PEF. By contrast, the application of the most intense treatments (8 kV/cm) caused a significant reduction in dry matter. This outcome could be explained by the fact that the application of PEF to a homogenate of fresh sage would facilitate the release of cellular compounds, including proteins, polysaccharides, and pigments, which may hypothetically interact and form insoluble complexes in hydroalcoholic solutions [43–45]. According to the literature, the occurrence of such interactions could reduce the soluble solids extracted into the hydroalcoholic solution [46–49]. For instance, chlorophyll molecules are known to interact with each other or with other compounds, leading to the formation of insoluble aggregates during extraction processes [50]. On the opposite, PEF treatment intensity did not significantly affect the dry matter content of the extract obtained from dried sage (Table 1). It can be hypothesized that this would result from the substantial cell collapse that occurred during drying, which might alter leaf tissue architecture (Figure 1B), potentially limiting the release of cellular components able to interact and form insoluble complexes [51, 52]. Table 1 also shows that the application of the most intense PEF treatment (8 kV/cm) resulted in an increase of both O.D. at 660 nm and total phenolic content of the extracts obtained from both fresh and dried sage. These results suggest that, regardless of the influence of the drying process on the tissue structure, these extraction conditions promoted the extraction of both chlorophylls and phenolic compounds (Figure 1).

To further explore the effect of PEF on the phytochemical profile of the extracts from fresh and dried sage, the relative abundance of the main phenol and chlorophyll‐related compounds identified by HPLC‐HRMS (Table S1) was assessed and reported in Table 2.

**Table 2:** Peak areas corresponding to the main phenol and chlorophyll‐related compounds detected in extracts obtained from fresh and dried sage by applying PEF treatments at increasing electrical field strengths (E). Controls indicate extracts not subjected to PEF.

Compound	Area x 10^7^ (counts^*^sec)					
	Fresh sage			Dried sage		
	E (kV/cm)			E (kV/cm)		
	0 (Control)	0.5	8	0 (Control)	0.5	8
Apigenin 7‐O‐glucuronide	7.56 ± 0.07^c^	8.76 ± 0.08^d^	7.65 ± 0.05^c^	11.12 ± 0.12^b^	11.46 ± 0.11^b^	12.22 ± 0.14^a^
Caffeic acid	115.27 ± 1.56^b^	125.92 ± 1.87^a^	114.93 ± 1.78^b^	79.93 ± 1.25^d^	83.32 ± 1.53^c^	85.85 ± 1.86^c^
Carnosic acid	22.27 ± 0.22^b^	24.33 ± 0.76^a^	22.36 ± 0.03^b^	13.44 ± 0.09^c^	13.34 ± 0.24^c^	12.54 ± 0.13^c^
Carnosol	982.19 ± 5.28^d^	1012.03 ± 5.45^c^	1016.50 ± 6.97^c^	1307.56 ± 6.25^b^	1310.37 ± 7.28^b^	1331.26 ± 8.34^a^
Cyranoside	32.21 ± 1.45^c^	37.27 ± 0.86^b^	39.00 ± 0.58^a^	17.18 ± 0.03^e^	28.87 ± 0.05^d^	31.27 ± 0.09^c^
Hydroxybenzoic acid	6.92 ± 0.06^d^	11.14 ± 0.04^b^	19.50 ± 0.19^a^	9.66 ± 0.11^c^	19.04 ± 0.89^a^	20.20 ± 0.94^a^
Luteolin 3‐O‐D‐glucuronide	35.84 ± 0.35^e^	38.66 ± 0.38^d^	23.16 ± 0.23^f^	45.57 ± 0.45^c^	47.57 ± 0.47^b^	50.51 ± 0.54^a^
Pelargonidin	4.33 ± 0.03^d^	5.82 ± 0.05^c^	4.88 ± 0.02^d^	13.86 ± 0.10^b^	16.04 ± 0.18^a^	16.64 ± 0.16^a^
Peonidin	30.69 ± 0.31^d^	41.80 ± 0.42^c^	42.50 ± 0.43^c^	54.59 ± 0.55^b^	64.25 ± 0.64^a^	65.17 ± 0.54^a^
Quercitrin	22.52 ± 0.65^d^	27.54 ± 0.75^c^	13.86 ± 0.54^e^	28.86 ± 0.78^b,c^	30.00 ± 0.84^b^	42.30 ± 0.27^a^
Rosmarinic acid	229.66 ± 3.45^f^	258.38 ± 2.58^e^	261.86 ± 2.62^d^	979.60 ± 6.79^c^	1036.90 ± 7.86^b^	1053.38 ± 8.94^a^
Salvianolic acid B	0.09 ± 0.01^d^	0.50 ± 0.56^c^	0.07 ± 0.01^d^	7.98 ± 1.46^b^	8.76 ± 2.04^a^	8.41 ± 1.87^a^
Salvianolic acid K	1.72 ± 0.57^e^	3.25 ± 1.25^d^	1.66 ± 0.42^e^	39.13 ± 1.43^c^	44.12 ± 1.85^b^	50.50 ± 2.01^a^
Chlorophyll A	0.30 ± 0.03^c^	0.46 ± 0.01^b^	1.45 ± 0.98^a^	0.08 ± 0.01^d^	0.08 ± 0.01^d^	0.10 ± 0.02^d^
Chlorophyll B	1.95 ± 0.08^b^	2.31 ± 0.12^a^	2.45 ± 0.16^a^	1.85 ± 0.09^b^	1.67 ± 0.04^c^	1.17 ± 0.02^d^
Chlorophyllide A	3.11 ± 1.05^c^	3.27 ± 1.01^c^	2.87 ± 0.97^d^	5.70 ± 1.04^a^	5.83 ± 1.27^a^	4.92 ± 0.96^b^
Chlorophyllide B	0.00 ± 0^c^	0.00 ± 0^c^	0.00 ± 0^c^	0.66 ± 0.01^b^	0.72 ± 0.01^a^	0.73 ± 0.01^a^
Pheophorbide A	0.67 ± 0.04^a^	0.63 ± 0.02^a^	0.64 ± 0.02^a^	0.42 ± 0.01^b^	0.42 ± 0.01^b^	0.42 ± 0.01^b^
Pheophorbide B	0.83 ± 0.07^b^	0.77 ± 0.06^b^	0.20 ± 0.02^c^	1.52 ± 0.14^a^	1.42 ± 1.04^a^	1.29 ± 1.05^a^
Pheophytin A	0.00 ± 0^d^	0.00 ± 0^d^	0.00 ± 0^d^	21.80 ± 1.06^c^	28.20 ± 1.65^a^	24.20 ± 0.96^b^
Pheophytin B	0.00 ± 0^c^	0.00 ± 0^c^	0.00 ± 0^c^	0.26 ± 0.27^b^	0.38 ± 0.32^b^	1.10 ± 1.16^a^

*Note:* Different letters within rows indicate statistically different values (*p* < 0.05).

**Figure 2:**
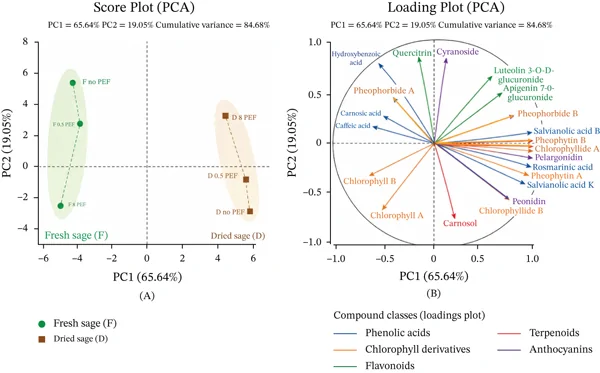
Principal component analysis (PCA) showing the distribution of compounds in extracts obtained from fresh and dried sage by applying PEF treatments at increasing electrical field strengths (E). Controls (F no PEF: fresh; D no PEF: dried) indicate extracts not subjected to PEF. (A) Score plot (PCA). (B) Loading plot (PCA).

In line with data reported in Table 1, the extracts from dried sage were generally richer in most identified phenolic compounds, whereas fresh sage extracts were generally richer in pigments contributing to the original green color of sage (chlorophylls, chlorophyllides, and pheophorbide) and did not contain those deriving from chlorophyll degradation, such as pheophytins. By contrast, the latter were clearly detected in the extracts obtained from dried sage. When PEF treatments with increasing intensity were applied to dried sage, an increase in the abundance of almost all phenol compounds was detected. To visualize how PEF treatments affected the composition of extracts from fresh and dried sage, a principal component analysis (PCA) was performed (Figure 2). PCA revealed that the first two principal components explained 84.68% of the total variance, with PC1 and PC2 accounting for 65.64% and 19.05%, respectively. PC1 represented the main source of variability and clearly separated fresh sage extracts from those obtained with the dried matrix. Variables showing the highest positive loadings on PC1 included salvianolic acid B (0.997), rosmarinic acid (0.994), salvianolic acid K (0.992), pelargonidin (0.991), pheophytin A (0.988), apigenin 7‐O‐glucuronide (0.973), chlorophyllide A (0.964), chlorophyllide B (0.946), and pheophorbide B (0.909), which were associated with dried sage extracts. In contrast, carnosic acid (−0.975), caffeic acid (−0.870), and chlorophyll B (−0.832) exhibited strong negative loadings and were associated with fresh sage extracts. These results further indicate that the drying process significantly influenced the qualitative phytochemical composition of sage extracts.

PC2 accounted for an additional 19.05% of the variance and was primarily influenced by cyranoside (0.957), quercitrin (0.930), and hydroxybenzoic acid (0.598), whereas chlorophyll A (−0.737) and carnosol (−0.709) contributed negatively. This component further differentiated samples according to variations induced by processing conditions, including PEF treatment intensity. Overall, the PCA suggests that the effect of drying was more pronounced than that of PEF treatment, whereas PEF intensity contributed to secondary changes in the relative abundance of specific phenolic and pigment‐related compounds. Extracts obtained from dried sage leaves treated at 8 kV/cm clustered near luteolin‐3‐O‐diglucuronide, rosmarinic acid, and several flavonoid derivatives, indicating that these PEF conditions particularly enhanced phenolic extraction. Conversely, extracts obtained from fresh leaves exposed to PEF—especially at 8 kV/cm—grouped closer to chlorophyll‐related vectors, whereas untreated samples from both matrices associated with lower phytochemical intensities. These differences can be attributed to the distinct structural characteristics of fresh and dried tissues and their corresponding responses to PEF. In fresh sage, the intact and turgid cells underwent extensive mechanical disruption during homogenization in the hydroalcoholic medium (Figure 1), resulting in efficient release of pigments, which was further slightly increased by subsequent PEF application [53]. In contrast, in dried sage, the drying process caused extensive collapse of cellular structures (Figure 1), which enhanced phenolic release during homogenization. Under these circumstances, PEF probably even further loosened the collapsed cellular matrix, enhancing solvent penetration and progressively increasing the solubilization of phenolic compounds [54].

The antioxidant activity of the samples was evaluated using DPPH, FRAP, and ABTS assays. To compare samples with different phenolic contents, the chain‐breaking phenolic ratio was also calculated (Table 3 **)**.

**Table 3:** Chain‐breaking phenolic ratio of extracts obtained from fresh and dried sage by applying PEF treatments at increasing electrical field strengths (E). Controls indicate extracts not subjected to PEF.

Sample	E (kV/cm)	Chain‐breaking phenolic ratio		
		DPPH (mg_TE_/mg_GAE_)	FRAP (mg_TE_/mg_GAE_)	ABTS (mg_TE_/mg_GAE_)
Fresh	0 (Control)	2.23 ± 0.17^b^	0.0021 ± 0.0005^c^	0.083 ± 0.08^b^
	0.5	2.46 ± 0.16^ab^	0.0036 ± 0.0001^b^	0.097 ± 0.11^ab^
	8	2.48 ± 0.06^a^	0.0045 ± 0.0001^a^	0.112 ± 0.03^a^

Dried	0 (Control)	1.76 ± 0.04^b^	0.0031 ± 0.0002^b^	0.022 ± 0.01^c^
	0.5	1.88 ± 0.14^ab^	0.0040 ± 0.0001^a^	0.029 ± 0.01^b^
	8	1.92 ± 0.03^a^	0.0041 ± 0.0005^a^	0.047 ± 0.09^a^

*Note:* Different letters indicate statistically different mean values in the same column (*p* < 0.05).

In fresh sage, the DPPH phenolic ratio increased from 2.23 to 2.48 mg TE/mg GAE as PEF intensity rose from 0 to 8 kV/cm, indicating that PEF slightly enhanced the radical scavenging efficiency of the extracted phenolics. A similar trend was observed for the FRAP phenolic ratio, which more than doubled (from 0.0021 to 0.0045 mg TE/mg GAE), suggesting an improvement in the reducing capacity of phenolic constituents. The ABTS phenolic ratio also increased from 0.083 to 0.112 mg TE/mg GAE at the highest PEF intensity, further confirming the enhanced chain‐breaking potential of phenolics released from fresh sage leaves under stronger electric fields. In dried sage extracts, the effect of PEF was more moderate but still consistent across assays. The DPPH phenolic ratio rose from 1.76 to 1.92 mg TE/mg GAE, whereas the FRAP ratio increased slightly from 0.0031 to 0.0041 mg TE/mg GAE. The ABTS phenolic ratio showed a comparable upward trend (from 0.022 to 0.047 mg TE/mg GAE), indicating that PEF also improved the chain‐breaking efficiency of phenolics in dried tissues, though to a lesser extent than in fresh leaves, suggesting that drying of the leaves plays a role in affecting the activity of phenolic compounds [55]. Overall, the results demonstrate that applying higher PEF intensities generally enhanced the antioxidant efficiency of phenolic compounds in both fresh and dried sage, with fresh tissue showing the most pronounced improvement across all assays. This behavior is consistent with the PEF‐induced facilitation of phenolic release in the extraction solvent (Table 2).

### 3.2. Effect of PEF on the Antimicrobial Properties of Sage Extracts

**Figure 3:**
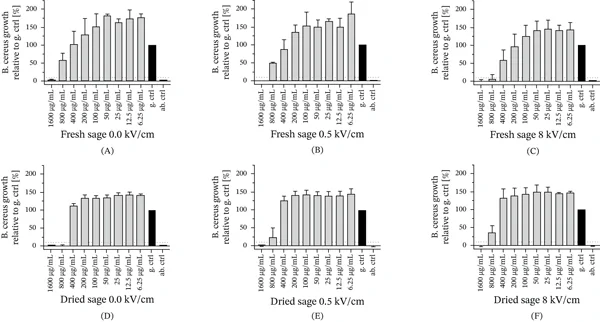
Relative growth of *B. cereus* in the presence of increasing concentrations of extracts obtained from (A–C) fresh and (D–F) dried sage by applying PEF treatments at increasing electrical field strengths (0, 0.5, and 8 kV/cm). G. ctrl and ab. ctrl indicate growth and positive antibiotic controls, respectively. Bars represent the mean of three independent experiments performed in triplicate (*n* = 3). Error bars indicate standard deviation. Dashed line indicates MIC_90_.

**Figure 4:**
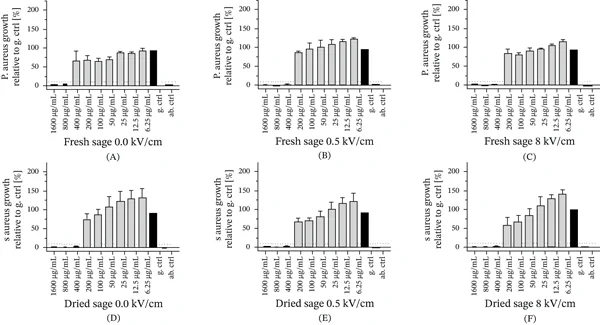
Relative growth of *S. aureus* in the presence of increasing concentrations of extracts obtained from (A–C) fresh and (D–F) dried sage by applying PEF treatments at increasing electrical field strengths (0, 0.5, and 8 kV/cm). G. ctrl and ab. ctrl indicate growth and positive antibiotic controls, respectively. Bars represent the mean of three independent experiments performed in triplicate (*n* = 3). Error bars indicate standard deviation. Dashed line indicates MIC_90_.

**Figure 5:**
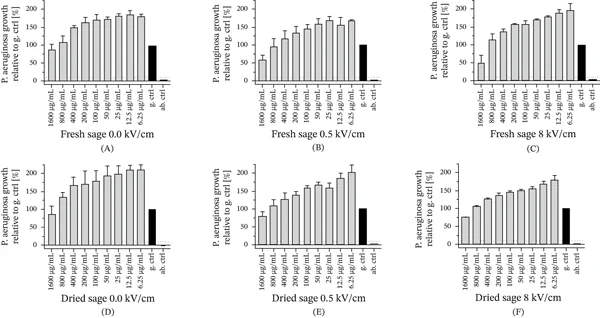
Relative growth of *P. aeruginosa* in the presence of increasing concentrations of extracts obtained from (A–C) fresh and dried sage and (D–F) dried sage by applying PEF treatments at increasing electrical field strengths (0, 0.5, and 8 kV/cm). G. ctrl and ab. ctrl indicate growth and positive antibiotic controls, respectively. Bars represent the mean of three independent experiments performed in triplicate (*n* = 3). Error bars indicate standard deviation. ns, *p* ≥ 0.05; ^*^ *p* < 0.05; ^**^ *p* < 0.01; ^***^ *p* < 0.001; ^****^ *p* < 0.0001.

The antimicrobial potential of fresh and dried sage extracts was assessed against five clinically relevant gram‐positive and gram‐negative bacterial strains: *B*. *cereus*, *S*. *aureus*, *P*. *aeruginosa*, *E*. *coli*, and *S*. *enterica.* The results of microbial growth trials and derived MIC_50_ and MIC_90_ values are shown in Figures 3, 4, and 5 and Tables 4 and S2.

**Table 4:** Minimum inhibitory concentrations (MIC) of extracts obtained from fresh and dried sage by applying PEF treatments at increasing electrical field strengths (E) required to inhibit 50% (MIC_50_) or 90% (MIC_90_) growth of *B. cereus*, *S. aureus*, and *P. aeruginosa.* Controls indicate extracts not subjected to PEF.

	Extract	E (kV/cm)	MIC_90_ (*μ*g/mL)	MIC_50_ (*μ*g/mL)
*B. cereus*	Dried	0 (Control)	800	800
		0.5	1600	800
		8	1600	800
	Fresh	0 (Control)	1600	800
		0.5	1600	800
		8	800	800

*S. aureus*	Dried	0 (Control)	400	400
		0.5	400	400
		8	400	400
	Fresh	0 (Control)	800	800
		0.5	400	400
		8	400	400

*P. aeruginosa*	Dried	0 (Control)	N.D.	N.D.
		0.5	N.D.	N.D.
		8	N.D.	N.D.
	Fresh	0 (Control)	N.D.	N.D.
		0.5	N.D.	N.D.
		8	N.D.	1600

*Note:* N.D.: not determined since no MIC was found.

All sage extracts reduced the growth of both gram‐positive bacteria, *B. cereus* and *S. aureus*, at concentrations ranging from 1600 to 400 *μ*g/mL. These strains were more susceptible to the dried extracts than to the fresh ones, with the exception of *B. cereus* at 8 kV/cm, which showed a growth inhibition of 90% (MIC_90_) at 1600 *μ*g/mL for the dried extract versus 800 *μ*g/mL for the fresh extract. Among the gram‐negative strains, only *P*. *aeruginosa* showed partial susceptibility, with a MIC_50_ of 1600 *μ*g/mL, against fresh sage extract processed at 8 kV/cm. All the other extracts failed to inhibit the growth of *P*. *aeruginosa* below 50% within the tested concentration range. For the fresh sage extracts, antimicrobial activity generally increased with higher PEF extraction intensity. In the dried extracts, however, extraction intensity showed no consistent effect on the inhibition of *S. aureus*, whereas for *B*. *cereus* higher intensities were associated with reduced growth inhibition. The markedly higher susceptibility of gram‐positive bacteria against the sage extracts aligns with previous findings [56]. Gram‐positive bacteria possess a thick peptidoglycan layer but only a single cytoplasmic membrane, which allows bioactive compounds such as phenolic acids to interact more readily with cellular targets [57, 58]. The overall higher phenolic content of the dried sage extracts can lead to a more pronounced bacteriostatic effect on gram‐positive bacteria than that observed with the fresh extracts. Higher concentrations of rosmarinic acid and carnosol in the dried extracts compared with the fresh extracts (Table 2) are likely key contributors to the stronger inhibition of *S. aureus* and *B*. *cereus* observed [59]. The enhanced antimicrobial activity of the fresh sage extracted at 8 kV/cm may be related to its higher content of diterpenes, particularly carnosol and carnosic acid (Table 2), which, together with chlorogenic acid has been reported to interfere with *P. aeruginosa* virulence mechanisms. Both compounds have been shown to suppress efflux pump activity and inhibit biofilm formation, providing a plausible mechanistic explanation for the improved antibacterial performance in the presence of other antimicrobial compounds [60, 61]. None of the extracts reached MIC_50_ or MIC_90_ values against *E. coli* or *S. enterica* (Figures S1 and S2), demonstrating that these species were resistant to the tested concentrations. Gram‐negative bacteria contain an additional outer membrane enriched with lipopolysaccharides, forming a hydrophobic and selectively permeable barrier that limits the penetration of many antimicrobial phytochemicals. This structural feature contributes substantially to their generally higher resistance against plant‐derived antimicrobial molecules [57, 58]. Overall, clear differences between fresh and dried extracts under different extraction conditions were observed. However, the relatively high concentrations required for microbial inhibition warrant cautious interpretation of these findings, and further validation in real food systems is needed to assess their practical relevance for food preservation applications.

## 4. Conclusions

This study demonstrates that PEF treatment is an effective nonthermal strategy to enhance the extraction of phenolic compounds from both fresh and dried sage. Increasing electric field intensity resulted in measurable improvements in antioxidant performance and antimicrobial properties against some gram‐positive bacteria, such as *B. cereus* and *S. aureus*. Overall, the findings confirm that PEF can modulate plant cell permeability and facilitate the release of phenolics without compromising product quality, highlighting its potential as a sustainable, energy‐efficient technology for valorizing aromatic plants and optimizing their functional properties.

To consolidate these findings and enable their transfer past the laboratory scale, further attention should be paid to both matrix‐related aspects and technical hurdles. At the matrix level, the structural state of the plant tissues subjected to extraction should be considered, as it may ultimately be relevant for a more accurate interpretation of the different extraction behaviors of fresh and dried matrices. On the other hand, at the technical level, it must be noted that a detailed and exhaustive framework of the energetic, environmental, and economic aspects of PEF‐assisted extraction from such matrices is still lacking. Filling this critical research gap would allow substantiating sustainability and commercial viability claims, paving the way for this technology up the TRL ladder and enabling further industrial applications and applied food research.

## Author Contributions

**Stella Plazzotta**: methodology, visualization, writing—original draft, and writing—review and editing; **Gian Marco Artuso**: formal analysis, investigation, writing—original draft, and writing—review and editing; **Sofia Melchior**: methodology, visualization, and writing—review and editing; **Federico Basso**: methodology, formal analysis, investigation, and writing—review and editing; **Sabrina Vorderegger**: methodology, investigation, formal analysis, writing—original draft, and writing—review and editing; **Monica Anese**: writing—review and editing; **Anja Schuster**: conceptualization, writing—original draft, supervision, resources, funding acquisition, and writing—review and editing; **Maria Concetta Tenuta**: methodology, software, formal analysis, investigation, and writing—review and editing; **Sara Bolchini**: methodology, software, formal analysis, investigation, and writing—review and editing; **Giovanna Ferrentino**: conceptualization, writing—original draft, supervision, resources, funding acquisition, and writing—review and editing; **Lara Manzocco**: conceptualization, funding acquisition, writing—original draft, supervision, resources, and writing—review and editing.

## Funding

The work was cofinanced by the European Union within the INTERREG Program IT‐AU (2021â€“2027) for the project entitled “Cross border cooperation for the valorization of alpine plants source of bioactive compoundsâ€“NETTLE (ITAT‐11‐007)”. Open access publishing was facilitated by the University of Udine, as part of the Wiley‐CRUI‐CARE agreement.

## Conflicts of Interest

The authors declare no conflicts of interest.

## Supporting information


**Supporting Information** Additional supporting information can be found online in the Supporting Information section. Table S1: Tentative identification by HPLC‐HRMS of chemical compounds present in extracts obtained from fresh and dried sage. Table S2: Statistical evaluation of relative growth of *B. cereus*, *S. aureus*, and *P. aeruginosa* in the presence of increasing concentrations of extracts obtained from fresh and dried sage by applying PEF treatments at increasing electrical field strengths (0, 0.5, and 8 kV/cm). ns, *p* ≥ 0.05; ^*^ *p* < 0.05; ^**^ *p* < 0.01; ^***^ *p* < 0.001; ^****^ *p* < 0.0001. Figure S1: Relative growth of *E. coli* in the presence of increasing concentrations of extracts obtained from (A–C) fresh and dried sage and (D–F) dried sage by applying PEF treatments at increasing electrical field strengths (0, 0.5, and 8 kV/cm). Figure S2: Relative growth of *S. enterica* in the presence of increasing concentrations of extracts obtained from (A–C) fresh and dried sage and (D–F) dried sage by applying PEF treatments at increasing electrical field strengths (0, 0.5, and 8 kV/cm).

## Data Availability

The data that support the findings of this study are available from the corresponding author upon reasonable request.
