# Supplementary material for: Effect of Pulsed Electric Field (PEF) Pretreatments on the Phytochemical Composition, Antioxidant, and Antimicrobial Properties of Sage Extracts
Source: Int J Food Sci. 2026 Sep 23;2026:3565398. doi: 10.1155/ijfo/3565398 (PMC13599367; doi:10.1155/ijfo/3565398)
Supplement: Supplementary file 1 [file IJFO-2026-3565398-s001.docx]

**Supplementary material**

**Table S1.** Tentative identification by HPLC-HRMS of chemical compounds present in extracts obtained from fresh and dried sage.

| Compound | RT | ESI | Experimental | Theoretical | Error | Formula | Fragments |
| --- | --- | --- | --- | --- | --- | --- | --- |
|  | **(min)** |  | **m/z** | **m/z** | **Ppm** |  |  |
| *Quinic acid* | 0.93 | [M-H]^-1^ | 191.0566 | 191.0561 | -0.0005 | C_7_H_12_O_6_ | 192.059, 127.040 |
| *Gallic acid* | 1.78 | [M-H]^-1^ | 169.0146 | 169.0141 | -0.0005 | C_7_H_6_O_5_ | 125.024, 97.029, 81.034, 69.034 |
| *Hydroxybenzoic acid* | 6.87 | [M-H]^-1^ | 137.0246 | 137.0244 | -0.0002 | C_7_H_6_O_3_ | 137.028, 93.034 |
| *Caffeic acid* | 9.68 | [M-H]^-1^ | 179.0353 | 179.035 | -0.0003 | C_9_H_8_O_4_ | 179.037, 135.040 |
| *Chlorogenic acid* | 10.96 | [M-H]^-1^ | 353.0885 | 353.0878 | -0.0007 | C_16_H_18_O_9_ | 191.053 |
| *Salvianolic acid A* | 11.06 | [M-H]^-1^ | 493.1143 | 493.114 | -0.0003 | C_26_H_22_O_10_ | 100.537, 295.433 |
| *Dihydrocaffeic acid* | 12.64 | [M-H]^-1^ | 181.0506 | 181.0513 | 0.0007 | C_9_H_10_O_4_ | 137.215, 109.145, 59.015 |
| *p-Coumaric acid* | 13.95 | [M-H]^-1^ | 163.0414 | 163.0401 | -0.0013 | C_9_H_8_O_3_ | 164.043, 119.058, 91.013 |
| *Dihydroxybenzoic acid glucuronide* | 15.85 | [M-H]^-1^ | 153.0194 | 153.0193 | -0.0001 | C_7_H_6_O_4_ | 109.029, 67.026 |
| *Ferulic acid* | 17.16 | [M-H]^-1^ | 193.0516 | 193.0506 | -0.001 | C_10_H_10_O_4_ | 178.028, 160.064, 134.041 |
| *Ellagic acid* | 19.09 | [M-H]^-1^ | 300.9991 | 300.9986 | -0.0005 | C_14_H_6_O_8_ | 257.215, 185.024 |
| *Hyperoside* | 19.26 | [M-H]^-1^ | 463.0882 | 463.0882 | 0 | C_21_H_20_O_12_ | 301.035, 300.467, 271.754 |
| *Quercitrin* | 20.24 | [M-H]^-1^ | 447.0937 | 447.0933 | -0.0004 | C_21_H_20_O_11_ | 271.032, 241.034 |
| *Cyranoside* | 20.26 | [M+H]^+1^ | 449.1081 | 449.1081 | 0 | C_21_H_20_O_11_ | 287.053, 288.058, 289.052 |
| *Luteolin 3-O-D-glucuronide* | 20.57 | [M-H]^-1^ | 461.0736 | 461.0726 | -0.001 | C_21_H_18_O_12_ | 285.046, 271.059 |
| *Rosmarinic acid* | 20.60 | [M-H]^-1^ | 359.0737 | 359.0772 | 0.0035 | C_18_H_16_O_8_ | 359.077, 197.045, 161.024, 135.045 |
| *Salvianolic acid K* | 21.61 | [M-H]^-1^ | 555.1151 | 555.1149 | -0.0002 | C_27_H_24_O_13_ | 359.077, 295.061, 179.035, 161.024, 135.045 |
| *Lithospermic acid* | 22.66 | [M-H]^-1^ | 537.1041 | 537.1038 | -0.0003 | C_27_H_22_O_12_ | 493.486, 295.063, 356.079 |
| *Apigenin 7-O-glucuronide* | 22.79 | [M-H]^-1^ | 445.0787 | 445.07763 | -0.00107 | C_21_H_18_O_11_ | 269.056, 113.022 |
| *Cyanidin* | 22.81 | [M+H]^+1^ | 287.0552 | 287.0546 | -0.0006 | C_15_H_11_O_6_ | 287.055, 288.058 |
| *Eryodictiol* | 23.02 | [M-H]^-1^ | 287.0563 | 287.0561 | -0.0002 | C_15_H_12_O_6_ | 151.003, 135.045, 255.028 |
| *Quercetin* | 23.08 | [M-H]^-1^ | 301.035 | 301.0354 | 0.0004 | C_15_H_10_O_7_ | 302.038, 273.040, 172.998 |
| *Hispidulin-7-O-D-glucuronide* | 23.54 | [M-H]^-1^ | 475.0882 | 475.0879 | -0.0003 | C_22_H_20_O_12_ | 299.056, 284.033 |
| *Luteolin* | 23.77 | [M-H]^-1^ | 285.0412 | 285.0405 | -0.0007 | C_15_H_10_O_6_ | 285.040, 223.027 |
| *Hesperidin* | 23.84 | [M-H]^-1^ | 609.1828 | 609.1825 | -0.0003 | C_28_H_34_O_15_ | 285.046, 136.082 |
| *Salvianolic acid B* | 24.13 | [M-H]^-1^ | 717.1472 | 717.1474 | 0.0002 | C_36_H_30_O_16_ | 719.166, 519.091, 475.103, 359.078, 229.014, 161.024 |
| *Apigenin* | 24.30 | [M-H]^-1^ | 269.0464 | 269.0455 | -0.0009 | C_15_H_10_O_5_ | 269.046, 221.118 |
| *Naringenin* | 24.95 | [M-H]^-1^ | 271.0615 | 271.0612 | -0.0003 | C_15_H_12_O_5_ | 271.0621, 272.060 |
| *Pelargonidin* | 25.17 | [M+H]^+1^ | 271.0614 | 271.0601 | -0.0013 | C_15_H_11_O_5_+ | 271.267, 273.162 |
| *Genistein* | 25.18 | [M-H]^-1^ | 269.0442 | 269.0455 | 0.0013 | C_15_H_10_O_5_ | 133.027, 159.034, 135.025 |
| *Malyngic acid* | 25.57 | [M-H]^-1^ | 327.2181 | 327.2177 | -0.0004 | C_18_H_32_O_5_ | 280.929, 96.964 |
| *Peonidin* | 25.66 | [M+H]^+1^ | 301.0713 | 301.0707 | -0.0006 | C_16_H_13_O_6_+ | 286.155 |
| *Jaceidin* | 26.08 | [M+H]^+1^ | 361.0926 | 361.0918 | -0.0008 | C_17_H_14_O_7_ | 300.061, 346.079 |
| *Malvidin* | 26.11 | [M+H]^+1^ | 331.0819 | 331.0812 | -0.0007 | C_17_H_15_O_7_+ | 270.037, 288.032 |
| *Jaceosidin* | 26.77 | [M+H]^+1^ | 331.0812 | 331.0823 | 0.0011 | C_17_H_14_O_7_ | 301.072 |
| *Carnosic acid* | 27.41 | [M-H]^-1^ | 331.1928 | 331.1915 | -0.0013 | C_20_H_28_O_4_ | 331.191, 287.201 |
| *Eupatorin* | 28.73 | [M+H]^+1^ | 345.0975 | 345.0969 | -0.0006 | C_17_H_14_O_6_ | 330.036, 312.122 |
| *Carnosol* | 31.92 | [M-H]^-1^ | 329.1761 | 329.176 | -0.0001 | C_20_H_26_O_4_ | 329.176, 285.186 |
|  |  |  |  |  |  |  |  |
| *Chlorophill A* | 11.20 | [M+H]^+1^ | 892.5337 | 892.5347 | 0.0010 | C₅₅H₇₂O₅N₄Mg | - |
| *Chlorophill B* | 11.60 | [M+H]^+1^ | 907.5214 | 907.5218 | 0.0004 | C_55_H_70_MgN_4_O_6_ | - |
| *Chlorophyllide A* | 12.14 | [M+H]^+1^ | 615.2459 | 615.2452 | -0.0007 | C_35_H_34_MgN_4_O_5_ | - |
| *Chlorophyllide B* | 12.61 | [M+H]^+1^ | 629.2265 | 629.2245 | -0.0020 | C_35_H_32_MgN_4_O_6_ | - |
| *Pheophorbide A* | 12.34 | [M+H]^+1^ | 593.2756 | 593.2759 | 0.0003 | C_35_H_36_N_4_O_5_ | - |
| *Pheophorbide B* | 12.27 | [M+H]^+1^ | 607.2550 | 607.2551 | 0.0001 | C_35_H_34_N_4_O_6_ | - |
| *Pheophytin A* | 16.00 | [M+H]^+1^ | 871.5735 | 871.5732 | -0.0003 | C_55_H_74_N_4_O_5_ | - |
| *Pheophytin B* | 15.47 | [M+H]^+1^ | 885.5522 | 885.5525 | 0.0003 | C_55_H_72_N_4_O_6_ | - |

**Table S2.** Statistical evaluation (Dunnet’s multiple pairwise comparisons test) of relative growth of *B. cereus*, *S. aureus and P. aueroginosa* in the presence of increasing concentrations of extracts obtained from fresh and dried sage by applying PEF treatments at increasing electrical field strengths (0, 0.5 and 8 kV/cm). ns p ≥ 0.05; * p < 0.05; ** p < 0.01; *** p < 0.001; **** p < 0.0001.

| ***B. cereus*** | | | | | | |
| --- | --- | --- | --- | --- | --- | --- |
| Pairwise comparison | Fresh 0.0 kV/cm | Fresh 0.5 kV/cm | Fresh 8 kV/cm | Dried 0.0 kV/cm | Dried 0.5 kV/cm | Dried 8 kV/cm |
| g. ctrl vs. 1600 µg/ml | *** | **** | **** | **** | **** | **** |
| g. ctrl vs. 800 µg/ml | ns | ns | **** | **** | **** | **** |
| g. ctrl vs. 400 µg/mL | ns | ns | ns | ns | * | * |
| g. ctrl vs. 200 µg/mL | ns | ns | ns | **** | ** | ** |
| g. ctrl vs. 100 µg/ml | ns | * | ns | **** | *** | ** |
| g. ctrl vs. 50 µg/ml | ** | ns | ns | **** | ** | *** |
| g. ctrl vs. 25 µg/ml | * | ** | * | **** | ** | *** |
| g. ctrl vs. 12.5 µg/ml | ** | ns | ns | **** | *** | ** |
| g. ctrl vs. 6.25 µg/ml | ** | *** | * | **** | *** | *** |
| ***S. aureus*** | | | | | | |
| Pairwise comparison | Fresh 0.0 kV/cm | Fresh 0.5 kV/cm | Fresh 8 kV/cm | Dried 0.0 kV/cm | Dried 0.5 kV/cm | Dried 8 kV/cm |
| g. ctrl vs. 1600 µg/ml | **** | **** | **** | **** | **** | **** |
| g. ctrl vs. 800 µg/ml | **** | **** | **** | **** | **** | **** |
| g. ctrl vs. 400 µg/mL | * | **** | **** | **** | **** | **** |
| g. ctrl vs. 200 µg/mL | * | ns | ns | ns | * | ** |
| g. ctrl vs. 100 µg/ml | ** | ns | ns | ns | * | * |
| g. ctrl vs. 50 µg/ml | * | ns | ns | ns | ns | ns |
| g. ctrl vs. 25 µg/ml | ns | ns | ns | ns | ns | ns |
| g. ctrl vs. 12.5 µg/ml | ns | * | * | * | * | ** |
| g. ctrl vs. 6.25 µg/ml | ns | ** | *** | ** | ** | **** |
| ***P. aueroginosa*** | | | | | | |
| Pairwise comparison | Fresh 0.0 kV/cm | Fresh 0.5 kV/cm | Fresh 8 kV/cm | Dried 0.0 kV/cm | Dried 0.5 kV/cm | Dried 8 kV/cm |
| g. ctrl vs. 1600 µg/ml | ns | * | *** | ns | ns | **** |
| g. ctrl vs. 800 µg/ml | ns | ns | ns | ns | ns | ns |
| g. ctrl vs. 400 µg/mL | *** | ns | ** | *** | * | **** |
| g. ctrl vs. 200 µg/mL | **** | ns | **** | *** | ** | **** |
| g. ctrl vs. 100 µg/ml | **** | ** | **** | **** | **** | **** |
| g. ctrl vs. 50 µg/ml | **** | *** | **** | **** | **** | **** |
| g. ctrl vs. 25 µg/ml | **** | **** | **** | **** | **** | **** |
| g. ctrl vs. 12.5 µg/ml | **** | *** | **** | **** | **** | **** |
| g. ctrl vs. 6.25 µg/ml | **** | **** | **** | **** | **** | **** |

**Figure S1.** Relative growth of *E. coli* in the presence of increasing concentrations of extracts obtained from fresh and dried sage (A-C) and dried sage (D-F) by applying MIPEF treatments at increasing electrical field strengths (0, 0.5 and 8 kV/cm). GC and PC indicate growth and positive controls. Bars represent the mean of three independent experiments performed in triplicate (n = 3). Error bars indicate standard deviation.

**Figure S2.** Relative growth of *S. enterica* in the presence of increasing concentrations of extracts obtained from fresh and dried sage (A-C) and dried sage (D-F) by applying MIPEF treatments at increasing electrical field strengths (0, 0.5 and 8 kV/cm). GC and PC indicate growth and positive controls. Bars represent the mean of three independent experiments performed in triplicate (n = 3). Error bars indicate standard deviation.
